# Predicting microRNA target genes using pan-cancer correlation patterns

**DOI:** 10.1186/s12864-025-11254-0

**Published:** 2025-01-27

**Authors:** Shuting Lin, Peng Qiu

**Affiliations:** 1 School of Biological Sciences, Georgia Institute of Technology, Atlanta, 30332 Georgia USA; 2 Department of Biomedical Engineering, Georgia Institute of Technology and Emory University, Atlanta, 30332 Georgia USA

**Keywords:** miRNA, Gene, Machine learning, TCGA

## Abstract

The interaction relationship between miRNAs and genes is important as miRNAs play a crucial role in regulating gene expression. In the literature, several databases have been constructed to curate known miRNA target genes, which are valuable resources but likely only represent a small fraction of all miRNA-gene interactions. In this study, we constructed machine learning models to predict miRNA target genes that have not been previously reported. Using the miRNA and gene expression data from TCGA, we performed a correlation analysis between all miRNAs and all genes across multiple cancer types. The correlations served as features to describe each miRNA-gene pair. Using the existing databases of curated miRNA targets, we labeled the miRNA-gene pairs, and trained machine learning models to predict novel miRNA-gene interactions. For the miRNA-gene pairs that were consistently predicted across the models, we called them significant miRNA-gene pairs. Using held-out miRNA target databases and a literature survey, we validated 5.5% of the predicted significant miRNA-gene pairs. The remaining predicted miRNA-gene pairs could serve as hypotheses for experimental validation. Additionally, we explored several additional datasets that provided gene expression data before and after a specific miRNA perturbation and observed consistency between the correlation direction of predicted miRNA-gene pairs and their regulatory patterns. Together, this analysis revealed a novel framework for uncovering previously unidentified miRNA-gene relationships, enhancing the collective comprehension of miRNA-mediated gene regulation.

## Introduction

MicroRNAs (miRNAs) are small, non-coding RNAs that play a crucial role in the post-transcriptional regulation of gene expression [1–3]. Typically, miRNAs silence target genes by binding to complementary sequences in the 3’ untranslated region of mRNAs, leading to their degradation and inhibiting their translation [4]. However, recent in vitro studies have suggested that miRNAs can also activate gene expression in certain circumstances through various mechanisms [5, 6]. Overall, miRNAs are versatile regulators of gene expression with complex roles in numerous biological processes, which has inspired many studies to explore the intricate relationship between miRNAs and their target genes [7, 8]. In recent years, extensive reviews have highlighted the pivotal role of miRNAs in human diseases. For example, miRNAs have been shown to regulate gene expression and are associated with a wide range of diseases, with computational models increasingly predicting miRNA-disease associations and integrating experimental data to better understand disease mechanisms [9]. Advances in bioinformatics tools and databases have further refined our knowledge of miRNA functions and their implications in human diseases. Systematic evaluations of these computational models have also demonstrated their potential to accurately predict miRNA-disease associations, enabling more comprehensive evaluations of miRNA functions in disease pathways [10]. Additionally, miRNAs are particularly studied for their regulatory roles in diseases such as cancer, where they significantly influence gene regulation and disease mechanisms [11]. Moreover, miRNAs modulate important signaling pathways involved in human diseases, such as PI3K-Akt and MAPK, making them attractive therapeutic targets [12]. Our study builds upon this foundation by predicting novel miRNA-gene interactions in cancer, contributing to the understanding of miRNA-mediated regulation in disease.

In the literature, researchers have determined many miRNA-gene relationships through experiments using high-throughput techniques on a transcriptome-wide scale, leading to a great number of publications and repositories of experimentally validated miRNA target genes [13–15]. For example, miRBase [16], DIANA-TarBase [17], and miRTarBase [18] are databases that provide comprehensive information about miRNAs and their targets. These databases include information on experimental methods and supporting literature to ensure the accuracy and reliability of the miRNA-gene interactions cataloged. In parallel to the experimental efforts, several computational approaches have been developed to predict miRNA-gene relationships [19–23]. TargetScan is one of the widely used methods that predict miRNA target sites conserved among orthologous 3’ UTRs of vertebrates based on the degree of sequence complementarity between the miRNA and its target mRNA [24]. While the recent developments in miRNA-gene databases have facilitated the exploration of miRNAs and provided new insights into their relationship with genes, these databases are not without limitations. Given the critical role of miRNA-gene regulation, the existing databases likely only represent a sparse coverage of miRNA-gene relationships, which provides an opportunity and motivation for this study, aiming to identify the yet unraveled miRNA-gene relationships.

In this study, we employed a machine learning approach to predict unknown miRNA-gene relationships by leveraging publicly available multi-omics gene expression data and several existing miRNA target databases. Using the miRNA and gene expression data from The Cancer Genome Atlas (TCGA) [25], we performed a comprehensive correlation analysis of each miRNA and each gene across 32 cancer types. We also compiled the known miRNA-gene relationships from the existing databases to label the miRNA-gene pairs. The total number of known miRNA-gene relationships in those databases is 197,877. After that, XGBoost was used to build machine learning models that predict potential miRNA-gene relationships that have not been reported in those miRNA target databases. In addition, our findings were further validated by held-out miRNA target databases and a literature survey, which showed the ability of the models in terms of predicting novel miRNA-gene relationships. Furthermore, by exploring independent datasets that contain gene expression before and after specific miRNA interventions, we found consistent alignment between the correlation direction of our predicted miRNA-gene pairs and their regulatory patterns shown in these independent datasets.

## Results

### Construction of miRNA-gene prediction models

To build models for predicting potential miRNA-gene relationships, we used the correlations between miRNA and gene expression data to serve as features for describing miRNA-gene pairs. TCGA provided expression data of a total of 1,881 miRNAs and 20,530 genes, for 10,004 patients across 32 cancer types. For each miRNA-gene pair, we computed their correlation in each of the 32 cancer types separately. Therefore, each miRNA-gene pair is described by a vector containing 32 correlation values. In order to avoid bias resulting from small sample sizes, for a miRNA-gene pair, their correlation in one cancer type was computed only when the number of patients expressing both the miRNA and the gene exceeded 10. We excluded miRNA-gene pairs whose correlations could not be calculated in more than three cancer types due to the limited sample sizes. As a result, we obtained the correlation values in the 32 cancer types for a total of 22,580,364 miRNA-gene pairs, involving 1,277 unique miRNAs and 17,822 unique genes.

The primary task of this study was to solve a binary classification problem to identify miRNA-gene association relationships. Binary class labels were needed to train the machine learning models. We gathered known miRNA-gene relationships from five existing databases of miRNA targets, including both experimentally validated and computationally predicted miRNA-gene interactions. These databases are miR2Disease [26], miRecords [27], TarBase [28], miRTarBase [18], and TargetScan [24]. We used mir2Disease, miRecords, and miRTarBase as training databases. The curated miRNA target genes in these training databases were used to define positive miRNA-gene pairs. The remaining two databases, TarBase and TargetScan served as validation databases. The miRNA target genes in the validation databases were used to evaluate the performance of our prediction. It is noteworthy that in evaluating model performance with the validation databases, we only considered those known miRNA-gene relationships that were only reported in the validation databases and not in the training databases.

For the 22,580,364 miRNA-gene pairs with correlation features computed, we labeled them as positives and negatives, depending on whether the miRNA-gene pair was reported in the training databases. Out of the 22,580,364 miRNA-gene pairs, 26,867 (0.12%) were labeled as positives because they were confirmed relationships in the training databases. The remaining 22,580,364 (99.88%) pairs were not present in the training databases, and thus, were labeled as negatives. Note that, due to the limited coverage of miRNA-gene interactions in the existing databases, the negative class included both unrelated pairs and true miRNA-gene relationships that were yet to be discovered.

**Fig. 1:**
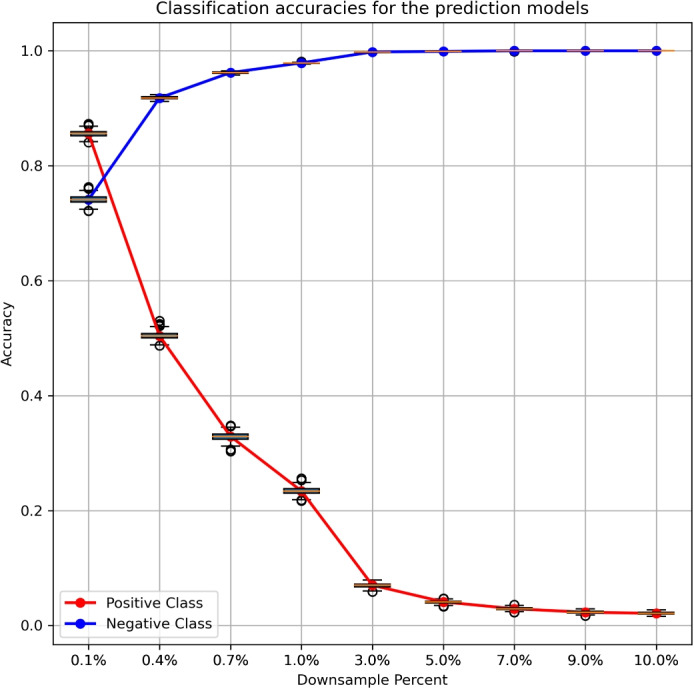
Classification accuracies for the prediction models on the testing datasets across 1000 experiments

To address the challenge of this extremely imbalanced binary classification task dominated by the negative class, we applied XGBoost (Extreme Gradient Boosting) to train machine learning models based on downsampled versions of the data. XGBoost is a powerful algorithm that excels in supervised classification problems [29]. Its ability to handle label-imbalanced data has been reported in many studies [30, 31], making it well-suited for identifying miRNA-gene relationships in our study. In addition, in order to reduce the imbalance, we generated many downsampled versions of the negative class to train XGBoost models. The size of the downsampled negative class ranged from 0.1% to 10% of the original negative class, so that the ratio between the positive and negative class after downsampling ranged from 1-to-1 to 1-to-100. At each downsampling level, we generated 1000 versions of downsampled negative class. We combined each of the downsampled negative class and the positive class, split the data with an 80/20 ratio of training and testing, trained a classifier using XGBoost, and evaluated testing performance for the positive and negative classes separately. Figure 1 shows the performance across the 1000 models trained based on the 1000 versions of downsampled data. As detailed in Fig. 1, incorporating more negatives into the training data led to a decreased prediction accuracy in the positive class (recall), while the prediction accuracy of the negative class (1-false positive) increased. This result was expected and suggested that a higher proportion of negatives might be more appropriate for identifying and hypothesizing miRNA-gene relationships for validation, because of the reduced false positives enabled by a higher proportion of negatives used in model training.

### Validation of the identified miRNA-gene pairs in the validation databases and literature

At each specified level of downsampling, we applied our suite of 1000 trained predictive models exclusively on the miRNA-gene pairs in the negative class. Our objective was to identify miRNA-gene pairs that were repeated identified as positive by these models, which may highlight potential miRNA-gene interactions previously undocumented in the literature. To ascertain the reliability of our findings, we concentrated on miRNA-gene pairs that were consistently classified as positive in at least 700 out of the 1000 applications across each downsampling threshold. We designated these frequently predicted pairs as ’significant miRNA-gene pairs’, thereby implying a higher confidence in their potential biological relevance. We evaluated the significant miRNA-gene pairs at different frequency requirements, including 700, 800, 900, and 950. Table 1 shows the number of identified miRNA-genes pairs at each downsampling level with various frequency requirements. As expected, with either increasing frequency requirement or increasing percentage of negatives, the number of significant miRNA-gene pairs decreased.

**Table 1:** Number of miRNA-gene pairs consistently identified as positives by predictive models with different frequency thresholds, and the proportion of these pairs reported in the validation database

Downsample				
Percent	700 times	800 times	900 times	950 times
0.1%	5036798/0.94%	4397542/0.97%	3457560/1.02%	2653818/1.08%
0.4%	1049382/1.29%	736636/1.34%	408392/1.41%	227812/1.49%
0.7%	338755/1.46%	214150/1.53%	104061/1.64%	53939/1.70%
1.0%	143100/1.62%	86201/1.66%	40217/1.77%	20554/1.82%
3.0%	8431/2.19%	5115/2.25%	2680/2.46%	1719/2.39%
5.0%	2857/2.52%	1948/2.72%	1220/2.79%	871/3.21%
7.0%	1683/2.91%	1217/2.96%	829/3.02%	630/3.02%
9.0%	1212/3.14%	913/3.07%	656/3.20%	496/3.02%
10.0%	1062/3.11%	816/2.82%	581/2.75%	434/2.76%

To evaluate the identified miRNA-gene pairs, we used two held-out miRNA target databases, which are the validation databases not included in the training and prediction process. The evaluation specifically focused on known miRNA-gene pairs that were reported in these validation databases but not in the training databases. For each downsample percentage and each frequency requirement, we computed the percentage of the identified miRNA-gene pairs that were reported in the validation databases but not reported in the training databases. As shown in Table 1, at downsample percentage of 5% and frequency requirement of 950, 871 miRNA-gene pairs were consistently predicted, which achieved the highest validation rate of 3.21%. Interestingly, we observed a similar validation rate for the 656 miRNA-gene pairs identified at downsample percentage of 9% and frequency requirement of 900. After comparing these two sets of the identified miRNA-gene pairs, we noticed that the 656 miRNA-gene pairs were a subset of the 871. Therefore, we decided to focus on the 871 miRNA-gene pairs in the following analyses.

Beyond the 28 (3.21% out of the 871) miRNA-gene pairs confirmed by the validation databases, there may exist more miRNA-gene pairs that have been discovered in the previous studies but were not included in the validation databases. Hence, we performed a literature survey for each of the remaining 843 miRNA-gene pairs. The PubMed database was used to search for previous publications that implicated the identified miRNA-gene pairs, and we found supportive literature for 20 miRNA-gene pairs, which were related to 5 miRNAs, including let-7b, mir-192, mir-26b, mir-335, and mir-93. For example, our predictions suggested a potential relationship between let-7b and EZH2, which aligns with a previous study demonstrating that the loss of let-7b leads to the upregulation of EZH2 expression, consequently promoting ovarian cancer growth both in vitro and in vivo [32]. Another example is miR-335 and TXNIP. A previous study has found that miR-335 targets several differential expressed genes, including TXNIP, and their interaction was associated with oral mucosal wound healing [33]. This previous study supported our prediction of the relationship between miR-335 and TXNIP. As a third example, mir-93 has been reported to control growth and proliferation through FOXO1, which served as a central regulator of endothelial activity [34]. Our analysis also indicates the potential association between mir-93 and FOXO1.

### Validation of the identified miRNA-gene pairs in independent datasets

To more rigorously confirm the link between the predicted miRNA-gene pairs, we conducted a thorough search for independent datasets regarding each of the miRNAs associated with the predicted miRNA-gene pairs that have not been previously documented in the miRNA targets databases. We searched for datasets that provided gene expression data both before and after perturbation of a specific miRNA, either overexpression or knockdown. As a result, we retrieved 9 GEO datasets, each designed to investigate the consequences after overexpressing a particular miRNA.

A total of 88 miRNA-gene pairs in different cancer contexts were validated in the independent datasets, which involved five miRNAs: mir-335, mir-192, mir-26b, mir-193b, and mir-21. Utilizing the gene expression data before and after a specific miRNA perturbation in each independent dataset, we performed differential expression analyses to identify the differential expressed genes (DEGs) that showed significant changes due to the miRNA perturbation. These genes may potentially be target genes for the miRNA. Each column of Table 2 corresponded to one independent dataset that investigated a particular miRNA. In each column of Table 2, we cataloged the number of identified DEGs from one independent dataset, the number of predicted miRNA-gene pairs we predicted for the miRNA, and the number of known target of the miRNA as documented in the five miRNA target databases. Additionally, we listed the intersection between the predicted targets and DEGs, along with the intersection between the known targets and DEGs. For example, in the existing literature, mir-335 has been associated with 3,411 target genes. Of these, 130 (4%) were detected as DEGs in the GSE68742 dataset. Among our predicted 569 target genes for mir-335, 31 (5%) were identified as DEGs. While a vast number of genes have been previously reported in the literature as targets of mir-335, only 4% of them manifested as differentially expressed genes (DEGs) in the GSE68742 dataset. Interestingly, our analysis, which predicted a smaller number of target genes, showed a slightly higher overlap of 5% with the DEGs. This observation was not an isolated occurrence. Upon extending this analysis for all 9 independent miRNA overexpression datasets, we found that our predicted miRNA target genes consistently demonstrated similar level of overlap with DEGs compared to the known target genes in the five miRNA target databases. This close alignment of overlap proportions indicated that our predictive method is potentially as robust as the broader literature in identifying miRNA targets.

**Table 2:** Comparison of predicted miRNA targets of different methods and known miRNA targets based on differentially expressed genes in independent datasets

miRNAs	mir-335	mir-335	mir-192	mir-192	mir-26b	mir-193b	mir-193b	mir-193b	mir-21
Dataset	GSE68742	GSE9586	GSE62951	GSE69990	GSE12091	GSE83690	GSE25215	GSE18510	GSE136665
DEGs	1162	1384	1377	1558	968	2108	1162	432	4677
Predicted targets	569	569	50	50	49	42	42	42	12
Known targets	3411	3411	1417	1417	1967	854	854	854	856
Overlap with DEGs:									
Our prediction	31 (5%)	26 (5%)	6 (12%)	4 (8%)	1 (2%)	7 (18%)	8 (20%)	2 (5%)	3 (25%)
PicTar	15 (11%)	17 (13%)	5 (10%)	4 (8%)	2 (4%)	9 (21%)	3 (7%)	0 (0%)	0 (0%)
RNA22	7 (6%)	6 (5%)	3.23 (6%)	3.23 (6%)	2.89 (6%)	4.25 (10%)	2.60 (6%)	0.89 (2%)	3.24 (27%)
miRanda	10 (2%)	2 (0%)	5 (10%)	3 (6%)	4 (8%)	0 (0%)	1 (2%)	0 (0%)	2 (17%)
TargetMiner	40 (7%)	32 (6%)	8 (16%)	2 (4%)	2 (4%)	5 (12%)	4 (10%)	1 (2%)	1 (8%)
PITA	37 (7%)	50 (9%)	6 (12%)	1 (2%)	5 (10%)	10 (24%)	5 (12%)	2 (5%)	3 (25%)
Known targets	130 (4%)	375 (11%)	87 (6%)	186 (13%)	154 (8%)	149 (17%)	98 (11%)	96 (11%)	259 (30%)

We also compared our model’s predictive performance against 5 established tools: PicTar [35], RNA22 [36], miRanda [37], TargetMiner [38], and PITA [39] using the 9 independent GEO datasets. For PicTar and RNA22, we assessed their performance using the precomputed predictions from the software platforms, while for miRanda, TargetMiner, and PITA, we computed scores for each miRNA-gene pair based on miRNA and 3’ UTR sequencing data from Ensembl [40] and evaluated their performance. Since our model’s predicted miRNA target genes consistently exhibited a similar overlap with DEGs as known targets, we extended this analysis to include predictions from other existing tools to evaluate whether their predicted targets also showed enrichment in DEGs within the GEO datasets. Specifically, for each of the five miRNAs in the independent datasets, we selected the top predicted targets from each tool, ensuring the number of selected targets matched the number of predictions generated by our model. We then calculated the overlap between these top predictions and the DEGs in each dataset. After that, we compared these overlaps among different methods across all the independent datasets. As shown in the last section of Table 2, all six methods achieved comparable levels of DEG enrichment across the 9 GEO datasets, aligning closely with the known targets. To examine whether the differences of the predictive performance among the methods are statistically significant, we performed paired t-tests on the overlap percentages (last section of Table 2) between each pair of methods. The *p*-values from these comparisons are shown in the Table 3. None of the *p*-values showed significance, suggesting that none of these methods outperformed the others. This is consistent with the observation that all methods achieve similar levels of DEG enrichment across all the independent datasets. In addition, we compared the predicted targets among different methods and found that each method identified distinct sets of targets, with only partial overlap. For the 5 miRNAs listed in Table 2, the shared targets between any two methods accounted for an average of 3.8% to 13.4% of the total predicted targets for each method. This pattern of low overlap underscores the variability in prediction outcomes across methods, and suggests that our approach complements existing tools by providing additional insights that may not be captured by sequence-based prediction methods alone.

**Table 3:** *P*-values from paired t-tests comparing DEG overlap percentages across predictive methods

	Our Prediction	PicTar	RNA22	miRanda	TargetMiner	PITA	Known Targets
Our Prediction	-	0.84	0.68	0.15	0.13	0.54	0.68
PicTar	0.84	-	0.80	0.14	0.11	0.63	0.51
RNA22	0.68	0.80	-	0.29	0.26	0.92	0.41
miRanda	0.15	0.14	0.29	-	0.87	0.15	0.06
TargetMiner	0.13	0.11	0.26	0.87	-	0.02	0.05
PITA	0.54	0.63	0.92	0.15	0.02	-	0.25
Known Targets	0.68	0.51	0.41	0.06	0.05	0.25	-

Moreover, the GSE68742 dataset explored the influence of mir-355 overexpression on gene expression within gastrointestinal stromal tumors (GISTs) [41], which are neoplasms closely related to SARC as delineated in the TCGA. As listed in the first column of Table 2, the overlap between the DEGs in GSE68742 and our potential mir-355 targets from TCGA is 31 genes. Among these 31 genes, 14 were up-regulated and 17 were down-regulated in the GSE68742 dataset. Since this dataset focused on overexpression of mir-355, the up-regulated genes are expected to have positive correlations with mir-355, while the down-regulated genes should exhibit negative correlations, especially within the context of SARC. We examined the observed correlation direction between the miRNA and these target genes in our TCGA analysis. Of the the 14 up-regulated DEGs, 6 exhibited the expected positive correlations with mir-355. Of the 17 down-regulated DEGs, 7 showed the expected negative correlations, as listed in the first section in Table 4. Such analysis of differential expression and correlation directions was conducted for each of the independent validation datasets. Overall, among the total of 88 miRNA-gene pairs (first overlap row in Table 2 and last column in Table 4) that showed differential expression in the validation datasets, we observed 40 whose directions of differential expression in the validation datasets agreed with their directions of miRNA-gene correlation in our TCGA analysis. These numbers translated to 45% of alignment between the differential expression after miRNA perturbation and the correlation in TCGA. Given the fact that TCGA data was generated from primary tumor samples while most of the independent datasets were generated from in vitro experiments, the 45% of alignment represented interesting miRNA target genes that exhibited robust regulatory patterns in different experimental designs, and can serve as hypotheses for further experimental validation.

**Table 4:** Examination of the relationship between the correlation direction of the predicted miRNA-gene pairs and their regulatory direction

miRNA	Datasets	Cancer context	Regulatory direction	Sign of correlation	Gene
mir-335	GSE68742	SARC	down	negative	CFLAR, FIGN, MSRB3, SOD3, RGS2, SGK1, PMP22
			down	positive	CCDC3, LDB2, DZIP1, KLF2, CXCL12, NTRK3, PABPC5, RBMS3, RCBTB2, RUNX1T1
			up	negative	A2M, ACTA2, DUSP3, TNFSF10, SGCB, SPARCL1, SSFA2, FAS
			up	positive	EGR2, JDP2, PRICKLE1, LOC728392, MATN2, LMO2
mir-335	GSE9586	LUAD	down	negative	CD247, DPYD, F3, LHFP, PTX3, MNDA, MT1E, MYL9, SCD5, SLFN11
			down	positive	OSR2
			up	negative	ADAM23, CALD1, CALHM2, CTTNBP2NL, DIXDC1, DUSP3, FBXL7, GCC2, GLI3, HVCN1, JAK1, MCTP1, SGCB, RHOJ, PCDHGA9
mir-192	GSE62951	LIHC	down	negative	E2F7, KIF2C, MTBP, NCS1, RAD54L
			up	positive	KIF4A
mir-192	GSE69990	OV	down	negative	SERTAD2
			down	positive	BIRC5, EZH2, PLK1
mir-26b	GSE12091	CESC	down	positive	C1S
mir-193b	GSE83690	SARC	down	positive	C9orf140, CCNB2, CENPA, KIF23, KIF2C, NUSAP1, TROAP
mir-193b	GSE25215	PAAD	up	positive	CCNB2, CDC25C, CENPO, KIF23, KIF2C, PTTG1, TROAP, TTK
mir-193b	GSE18510	SKCM	up	positive	BIRC5, KIF2C
mir-21	GSE136665	COAD	up	negative	ACADSB, NAP1L5
			up	positive	AKAP11

## Discussion

In this study, we aimed to identify potential miRNA-gene associations that have not been previously reported in the literature. Specifically, we used multi-omics data of cancer patients to compute correlate features for miRNA-gene pairs, leveraged existing miRNA target databases to define class labels, and applied XGBoost to train machine learning models to predict potential miRNA-gene relationships. Among the 871 predicted significant miRNA-gene pairs, 5.5% were validated using independent held-out miRNA target databases and literature survey, while the remaining could serve as promising hypotheses for future experimental validation. In addition, we further validated the identified miRNAs by analyzing gene expression profiles before and after specific miRNA perturbations in multiple independent datasets, and we consistently observed an alignment between the correlation direction of the predicted miRNA-gene pairs and their regulatory patterns presented in these datasets. Although there were instances where the correlation direction diverged from their regulatory patterns in the independent datasets, such discrepancies can be attributed to the fact that these validation datasets were derived from cell lines, while the correlations we calculated were based on primary tumor samples profiled by TCGA.

This study is not without limitations. A key issue is that the existing literature provides only a limited number of confirmed miRNA-gene interactions. The lack of data makes it difficult for machine learning models to identify all potential relationships due to the imbalance between confirmed interactions and unreported ones. This imbalance has significant impact on our machine learning model. When working with such imbalanced data that was dominated by the negative class, the model is biased toward predicting negatives. To address this, we performed data downsampling to reduce the prevalence of the negative class and trained our model on various downsampled versions of the data. By doing so, we ensured that the model could focus more on potential miRNA-target relationships rather than being overwhelmed by the prevalence of negative pairs. The imbalance also means that our approach cannot be expected to capture all possible miRNA-target interactions without generating a large number of false positives. Instead, our goal was to focus on identifying high-confidence predictions, which may serve as hypotheses for further experimental validation. As we continue to refine our models, incorporating additional datasets and experimental techniques may help improve the predictive power of our approach.

Nevertheless, our study introduced a new methodology to discover previously unreported miRNA-gene associations, advancing a comprehensive understanding of miRNA-gene interactions. The findings from this investigation offer insight into miRNA regulation and hypotheses for experimental explorations.

## Methods

### Data access

The miRNA expression data was downloaded from TCGA Genomic Data Commons (https://portal.gdc.cancer.gov/). A total of 1,881 miRNAs were included in this study. The miRNA expression data was subsequently log-transformed for analysis. The gene expression data were downloaded from ucsc xena (http://xena.ucsc.edu) (IlluminaHiSeq, level 3 expression), which comprised a total of 20,530 genes. The miRNA and gene expression data covered 10,004 patients across 32 cancer types (glioblastoma was excluded from this study due to the small sample size in miRNA data).

In addition, we collected established miRNA targets from five different databases (mir2Disease, miRecords, TarBase, miRTarBase, and TargetScan) to label each miRNA-gene pair during model training. In total, 197,877 known miRNA-gene relationships were included in this study.

For the purpose of validating the predicted miRNA-gene pairs, a total of 9 datasets were collected from the GEO database. These datasets were specifically selected to contain gene expression profiles both before and after specific miRNA perturbations. These datasets were GSE68742, GSE9586, GSE62951, GSE69990, GSE12091, GSE83690, GSE25215, GSE18510, and GSE136665.

To compare our method with other existing tools, such as, miRanda, TargetMiner, and PITA, we downloaded 924 human miRNA sequences and 15,705 3$'$ UTRs from Ensembl database (https://useast.ensembl.org/index.html) using BiomaRt tool.

### Correlation analysis between miRNAs and genes

We performed a Pearson’s correlation analysis between miRNA expression and gene expression for each cancer type. To mitigate the effect of small sample sizes, we calculated correlation coefficients only for miRNA-gene pairs with expression data from at least 10 patients per cancer type. Pairs that could not be calculated in more than three cancer types were excluded from the study. All statistical tests were performed using standard Python functions.

### Construction of prediction model

We used XGBoost (Python library version 2.1.1) to perform binary classification, predicting potential miRNA-gene relationships. The analysis involved over 22 million miRNA-gene pairs, each labeled as either positive or negative based on existing miRNA target databases. Only 0.12% of the pairs were labeled as positive, representing confirmed miRNA-gene relationships, while the remaining 99.88% were labeled as negative since they were not documented in the databases. To address this significant class imbalance, we downsampled the negative class at various rates (ranging from 0.1% to 10%). For each downsampled version of the data, samples (miRNA-gene pairs) in the data were split, with 80% used for training and 20% for testing. Model prediction performance was evaluated using the Area Under the Curve (AUC) metric. At each downsampling level, we trained 1000 models, which were applied only to the negative class. MiRNA-gene pairs consistently predicted by these models were identified as potential, previously undiscovered miRNA target relationships.

### Literature survey

A literature search was performed using PubMed. We programmatically searched the PubMed database using custom Python scripts. We searched through PubMed for all keywords in all fields, including title, abstract, and main texts of the articles.

### Differential expression analysis

In each of the independent datasets, we performed a t-test to identify genes that expressed differently before and after the specific miRNA perturbations. Genes were considered as DEGs if the significant *p*-value <0.05.

## Data Availability

All data used in this analysis can be found at the GDC data portal (https://portal.gdc.cancer.gov/), mir2Disease (http://mir2disease.org/), miRecords (http://c1.accurascience.com/miRecords/), TarBase (https://dianalab.e-ce.uth.gr/tarbasev9), miRTarBase (https://mirtarbase.cuhk.edu.cn/~miRTarBase/miRTarBase_2022/php/index.php), and TargetScan (https://www.targetscan.org/vert_80/). The code and data used for predicting miRNA targets are available on figshare with the link: https://figshare.com/projects/Predicting_miRNA_target_genes/223452.
